# Chronic treatment of non-small-cell lung cancer cells with gefitinib leads to an epigenetic loss of epithelial properties associated with reductions in microRNA-155 and -200c

**DOI:** 10.1371/journal.pone.0172115

**Published:** 2017-02-22

**Authors:** Michiko Narita, Eri Shimura, Atsumi Nagasawa, Toshiki Aiuchi, Yukari Suda, Yusuke Hamada, Daigo Ikegami, Chizuru Iwasawa, Kazuhiko Arakawa, Katsuhide Igarashi, Naoko Kuzumaki, Yusuke Yoshioka, Takahiro Ochiya, Hideyuki Takeshima, Toshikazu Ushijima, Minoru Narita

**Affiliations:** 1 Department of Pharmacology, Hoshi University School of Pharmacy and Pharmaceutical Sciences, Ebara, Shinagawa-ku, Tokyo, Japan; 2 Life Science Tokyo Advanced Research Center (L-StaR), Hoshi University School of Pharmacy and Pharmaceutical Sciences, Ebara, Shinagawa-ku, Tokyo, Japan; 3 Division of Molecular and Cellular Medicine, National Cancer Center Research Institute, Tsukiji, Chuo-ku, Tokyo, Japan; 4 Division of Epigenomics, National Cancer Center Research Institute, Tsukiji, Chuo-ku, Tokyo, Japan; University of Central Florida, UNITED STATES

## Abstract

**Background:**

The EGFR tyrosine kinase inhibitor gefitinib is used in therapy for non-small-cell lung cancer (NSCLC). However, its application is limited by resistance-accelerated disease progression, which is accompanied by the epithelial-to-mesenchymal transition (EMT). In the present study, we performed multiple expression analyses of microRNAs (miRNAs) and quantified the expression of several related EMT players in gefitinib-resistant NSCLC cells.

**Methods and results:**

To establish gefitinib-resistant NSCLC cells, gefitinib-sensitive HCC827 cells, which exhibit an in-frame deletion [E746-A750] in EGFR exon 19, were exposed to gefitinib for at least 1.5 months. Next, to profile “gefitinib-resistant HCC827 (HCC827GR)” cells, which have a secondary T790M mutation in EGFR exon 20, a miRNA array analysis was performed in HCC827 and HCC827GR cells. The greatest differences were seen in the levels of miR-155 and miR-200c, which essentially disappeared in HCC827GR cells. In addition to these reductions, the levels of smad2 and zeb1, which are both key players in EMT and targets for miR-155 and miR-200c, respectively, were dramatically increased in HCC827GR cells. In HCC827GR cells, the expression of epithelial-cadherin (E-cadherin) was greatly reduced with repressive histone modifications, whereas vimentin, which is expressed in mesenchymal cells, was dramatically increased with active histone modifications. In another gefitinib-resistant NSCLC cell line (H1975 cells), similar to the findings in HCC827GR cells, both miR-155 and miR-200c were absent, and the EMT was induced along with epigenetic modifications. Interestingly, the inhibition of both miR-155 and miR-200c in HCC827 cells without gefitinib induced significant increases in smad2 and zeb1 along with a dramatic decrease in E-cadherin and a slight increase in vimentin. Furthermore, although the inhibition of these miRNAs in HCC827 cells decreased gefitinib sensitivity, this dual-inhibition in HCC827 cells without gefitinib did not produce a secondary T790M mutation in EGFR exon 20.

**Conclusion and implications:**

These results suggest that chronic treatment of NSCLC cells with gefitinib changes the expression of miRNAs, including dramatic reductions in miR-155 and miR-200c along with an EGFR mutation. Furthermore, this depletion of miR-155 and miR-200c may be associated with the EMT along with histone modifications, and may contribute to the decrease in the sensitivity to gefitinib independent of a secondary EGFR mutation.

## Background

Cancer is the most common cause of death, and lung cancer is the leading cause of death from cancer. Among the different forms of lung cancer, non-small-cell lung cancer (NSCLC) is treated with an epidermal growth factor receptor (EGFR) tyrosine kinase inhibitor, such as gefitinib [1]. EGFR is commonly overexpressed or aberrantly active in NSCLC. Activation of the EGFR provides signals that drive dysregulated proliferation, invasion, metastasis, angiogenesis, and cell survival, and its inhibition has potential for both the treatment and prevention of these malignancies [2]. However, the application of gefitinib is ultimately limited by the emergence of acquired drug resistance, which is mainly mediated by a secondary T790M mutation in EGFR [3, 4]. Furthermore, acquired resistance to gefitinib is associated with a clinically significant risk of accelerated disease progression [5], which is also accompanied by the epithelial-to-mesenchymal transition (EMT).

On the other hand, epigenetic modifications, such as DNA methylation, histone modifications, and the expression of noncoding RNA such as microRNAs (miRNAs), have recently been widely reported to play a major role in diseases including cancer [6]. Above all, increasing interest has been focused on the role of miRNAs in cancer. miRNAs are noncoding RNAs of 19–24 nucleotides that mainly bind to the 3’UTRs of mRNAs and regulate their expression post-transcriptionally. In addition, a single miRNA can target scores of mRNAs, and thereby control a wide range of biological functions. Changes in miRNA expression have been observed in cancers in various tissues such as lung [7], breast [8], liver [9], colon and rectum [10] and prostate [11]. Aberrantly expressed miRNAs exert their functions by modulating oncogenic or tumor-suppressing genes and play important roles in the development, progression and drug-resistance of cancers.

In the present study, we performed multiple analyses of the expression of miRNAs in gefitinib-resistant NSCLC cells. Furthermore, we investigated how changes in miRNAs are associated with the EMT through epigenetic modifications in gefitinib-resistant NSCLC cells.

## Materials and methods

### Reagent

The reagent used in the present study was the EGFR tyrosine kinase inhibitor N-(3-chloro-4-fluoro-phenyl)-7-methoxy-6-(3-morpholin-4-ylpropoxy)-quinazolin-4-amine (gefitinib; Toronto Research Chemicals Inc., ON, Canada).

### Establishment of the gefitinib-resistant NSCLC cell line HCC827GR

The human NSCLC cell line HCC827 (American Type Culture Collection Co., MD, USA) was exposed to 1 μM of gefitinib for 48 h in RPMI-1640 Medium HEPES Modification (Sigma-Aldrich Co., MO, USA) containing 10% fetal bovine serum (FBS; Invitrogen^™^ Thermo Fisher Scientific Inc., MA, USA) and 1% penicillin-streptomycin (P/S; Invitrogen^™^ Thermo Fisher Scientific Inc.). The cells were then washed and cultured in drug-free medium until surviving cells were 80% confluent. These cells were then re-exposed to increasing concentrations of gefitinib, from 1 to 5 μM. Cells that were finally able to grow in 5 μM gefitinib were obtained 1.5 months after the initial exposure as described previously [12]. The established resistant cells were maintained in medium containing 1 μM of gefitinib. For all in vitro studies, resistant cells were eventually cultured in drug-free medium for at least 1 week to eliminate gefitinib. All cells were maintained under a humidified atmosphere of 5% CO_2_ at 37°C. Gefitinib-resistant HCC827 cells are referred to here as HCC827GR cells.

### TaqMan miRNA arrays and real-time PCR for miRNA

Total RNA, including miRNAs extracted from HCC827 and HCC827GR cells, was reverse-transcribed using a TaqMan miRNA Reverse Transcription (RT) kit (Applied Biosystems^®^ Life Technologies Co., MA, USA) in combination with the stem-loop Megaplex primer pool (Applied Biosystems Thermo Fisher Scientific Inc.). miRNA expression profiles were acquired using the TaqMan^®^ Array MicroRNA Card for Human (Applied Biosystems Thermo Fisher Scientific Inc.). Quantitative RT-PCR was performed at Life Technologies Japan Ltd. using an Applied Biosystems 7900HT Fast Real-Time PCR system. Normalized expression (NE) was calculated as NE = 2^-ΔΔ^*C*t, where *C*t is the threshold cycle for detecting fluorescence. The data were normalized to U6 snRNA. Individual TaqMan assays were performed using the Single Tube TaqMan^®^ MicroRNA assay (Applied Biosystems Thermo Fisher Scientific Inc.) following the manufacturer's protocol for the expression of miR-155 and miR-200c in HCC827 and HCC827GR miRNAs as described previously [13]. All primers are listed in S1 Table.

### miRNA target prediction

miRNA-targeting predictions were queried in three target-prediction databases: TargetScan (http://www.targetscan.org), PITA (http://genie.weizmann.ac.il/pubs/mir07) and DIANA-microT (http://diana.pcbi.upenn.edu/cgi-bin/microt.cgi).

### Sample preparation and western blotting

HCC827 or HCC827GR cells were solubilized with buffer containing 20 mM Tris-HCl (pH7.4), 0.3%(w/v) Triton, 3 mM MgCl_2_, 1 M sucrose, 5 mM α-ME, and 1/1,000 protease inhibitor or M-PER Mammalian Protein Extraction Reagent (Thermo Fisher Scientific Inc.), and cell lysates were prepared as described previously [12]. For immunoblot detection, a nitrocellulose membrane was incubated with the following primary antibodies: anti-smad family member 2 (smad2; Cell Signaling Technology Inc., MA, USA), anti-phospho smad2 (p-smad2; Cell Signaling Technology Inc.) anti-zinc finger E-box binding homeobox 1 (zeb1; Santa Cruz Biotechnology, TX,USA), anti-epithelial cadherin (E-cadherin; Becton Dickinson Co., NJ, USA), anti-vimentin (Cell Signaling Technology Inc.), anti-glyceraldehyde-3-phosphate dehydrogenase (GAPDH; Merck Millipore Co., MA, USA) and anti-fibrillarin (Abcam, Cambridge, UK). The membrane was then incubated with secondary antibody and detected by enhanced chemiluminescence (Pierce, IL, USA) with visualization by exposure to Amersham Hyperfilm (Amersham Life Sciences, IL, USA). All antibodies are listed in S2 Table.

### Dual luciferase 3’UTR-reporter assays

For the luciferase assay, dual luciferase reporter vectors (psiCHECK-2; Promega Co., WI, USA) that contained either the 3’UTR of smad2, the 3’UTR of zeb1, mutant smad2 or mutant zeb1 were used as described with the primers listed in S3 Table. HEK293 cells were cotransfected with reporter vectors and expression vectors of miR-155, pre-miR-200c mimics (Invitrogen^™^ Thermo Fisher Scientific Inc.) or pre-miR^™^ miRNA Precursor Negative Control #1 (miR-NC; Invitrogen^™^ Thermo Fisher Scientific Inc.) using DharmaFECT Transfection Reagents (Thermo Fisher Scientific Inc., MA, USA). The activities of renilla and firefly luciferase were quantified with the Dual Luciferase Reporter Assay System (Promega Co.) according to the manufacturer’s protocol. Luminescent signal was quantified by a luminometer (Glomax, Promega Co.), and, for normalization of differences in transfection, renilla luciferase activity was related to its firefly counterpart.

### Real-time PCR for mRNAs

One microgram of purified RNA was used for a subsequent real-time PCR performed as described previously [12] with the synthesized primers for smad2, zeb1, E-cadherin, vimentin and GAPDH, which are shown in S4 Table.

### Immunocytochemistry: Staining of cells with E-cadherin and vimentin

HCC827 and HCC827GR cells were fixed in 4% paraformaldehyde. After being blocked in 0.3% Triton with 5% FBS, cells were incubated with either anti-E-cadherin or vimentin as a primary antibody. The primary antibody was then rinsed away and cells incubated with an appropriate secondary antibody conjugated with Alexa 488 or Alexa 546. The slides were cover-slipped with Dapi fluoromount-G (Southern Biotech, Birmingham, AL, USA). Cells were observed with a laser scanning confocal microscope (FV1200, Olympus, Tokyo, Japan). All antibodies are listed in S2 Table.

### Wound healing assay

HCC827 and HCC827GR cells were placed in both wells of a Culture Insert 2 Well (ibidi, Munich, Germany). After cells were incubated at 37°C under 5% CO_2_ for 24 hr, they were gently removed from the culture insert according to the manufacturer’s protocol, observed a microscope (Olympus CKX41, Olympus) and photographed with a digital camera (Digital Sight, Nikon, Japan).

### Chromatin Immunoprecipitation (ChIP)

A chromatin immunoprecipitation assay was performed as described previously [14] with minor modifications. HCC827 or HCC827GR cells were first cross-linked with 1% formaldehyde and then treated with 2.5M glycine before being washed with 1×PBS(-). Cells were re-suspended in lysis buffer (50 mM Tris-HCl at pH 8.0, 1 mM EDTA, 1% SDS), incubated and then sonicated to shear DNA with a Picoruptor ver1 (Diagenode Inc., NJ, USA). After DNA shearing, the lysate was centrifuged and supernatant was recovered. Fifteen μg of sheared DNA was immunoprecipitated with 2 μg of specific antibodies against acetylated histone H3 (AcH3; Merck Millipore Co.), histone 3 trimethylated at Lys4 (H3K4me3; Wako Pure Chemicals, Japan), Lys9 (H3K9me3; Merck Millipore Co.), or Lys27 (H3K27me3; Merck Millipore Co.), and Dynabeads^®^ Protein A (Thermo Fisher Scientific Inc., MA, USA). Sheared chromatin (20 μL) was recovered as input DNA. After the reversal of cross-linking, DNA was recovered by treatment with RNase A and proteinase K refined by phenol/chloroform extraction and isopropanol precipitation, and dissolved in 30 μL of 1xTE. One μL of immunoprecipitated and input DNA was used for ChIP-quantitative PCR (ChIP-qPCR). ChIP-qPCR was performed as described with the primers listed in S5 Table. All antibodies are listed in S2 Table.

### Inhibition of miR-155 and miR-200c in HCC827 cells

Specific inhibitors of miR-155 and miR-200c and a negative control were purchased from Ambion Thermo Fisher Scientific Inc. HCC827 cells were co-transfected with specific miR-155 and miR-200c inhibitors (miR-155/200c IH), or mirVana^™^ miRNA Inhibitor, Negative Control #1 (NC; Ambion Thermo Fisher Scientific Inc.) using DharmaFECT Transfection Reagents (Thermo Fisher Scientific Inc.).

### Statistical analysis

The statistical significance of differences between groups was assessed by one-way analysis of variance (ANOVA) followed by the Bonferroni/Dunn multiple comparison test. The statistical significance of differences between two groups was assessed with Student’s *t*-test.

## Results

### Effects of gefitinib on the growth of gefitinib-sensitive NSCLC cells (HCC827) and gefitinib-resistant NSCLC cells (HCC827GR)

Addition of the tyrosine kinase inhibitor gefitinib (0.001 μM-0.1 μM) to HCC827 cells, which are considered to be a gefitinib-sensitive NSCLC cell line, for 2 days produced a concentration-dependent decrease in tumor cell growth (S1A Fig, p<0.001 vs. non-treated group). A sequence analysis confirmed that HCC827 cells had an in-frame deletion [E746-A750] in EGFR exon 19, which is known to be highly sensitive to EGFR tyrosine kinase inhibitor (S1B Fig).

To establish gefitinib-resistant NSCLC cells, HCC827 cells were exposed to 1 to 5 μM of gefitinib for at least 1.5 months. Gefitinib-resistant HCC827 cells are referred to here as HCC827GR cells. Treatment with gefitinib (0.001 μM-0.1 μM) for 2 days did not affect the growth of HCC827GR cells (S1C Fig). According to a sequence analysis, HCC827GR cells had a second point mutation, which was believed to be mainly responsible for drug resistance: a nucleotide 2369 C→T mutation in EGFR exon 20, which led to the transition Thr790Met (S1D Fig).

### Changes in miRNA expression in HCC827GR cells

**Fig 1:**
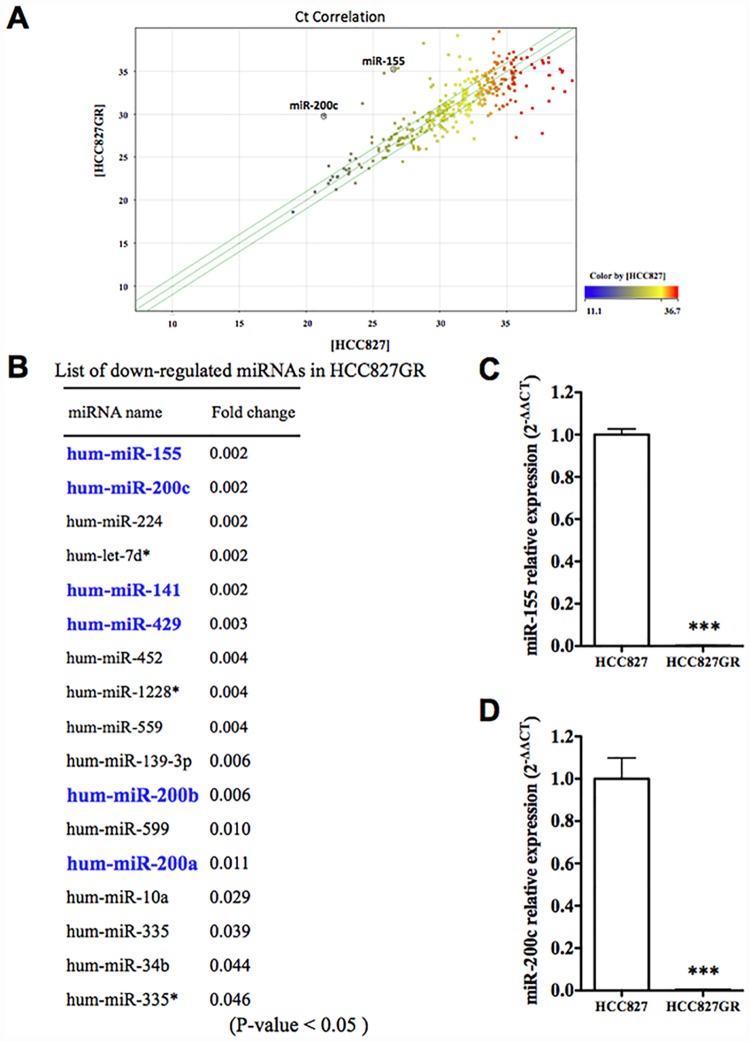
miRNA expression analysis using microarrays. (A) Pearson’s correlation scatter plot of miRNA levels between HCC827 and HCC827GR cells. (B) List of down-regulated miRNAs in HCC827GR cells compared to HCC827 cells (fold change of p<0.05). (C,D) qRT-PCR for miR-155, miR-200c and RNU44, an internal standard, in HCC827 cells and HCC827GR cells (***p<0.001 vs. HCC827 cells).

To profile the expression of mRNAs encoding 768 miRNAs in HCC827 and HCC827GR cells, a miRNA array analysis was performed. The scatter plot showed that there were differences in the expression of miRNA, and there were many more down-regulated miRNAs than up-regulated miRNAs in HCC827GR cells compared to HCC827 cells (Fig 1A). Among the down-regulated miRNAs, the expressions of miR-155, miR-200c, miR-224, let-7d* and miR-141 were dramatically decreased in HCC827GR cells. The expressions of miR-429, miR-452, miR-1228*, miR-559, miR-139-3p, miR-200b, miR-599, miR-200a, miR-10a, miR-335, miR-34b and miR-335* were also decreased (Fig 1B, fold change <0.05) (*: The complementary strand of a nascent duplex microRNA molecule). To confirm these results of the miRNA array analysis, a real-time PCR analysis of miR-155 and miR-200c, which were the most strongly down-regulated miRNAs, was conducted in HCC827 and HCC827GR cells. Consistent with the results of the miRNA array analysis, the expression levels of miR-155 and miR-200c were significantly decreased in HCC827GR cells compared to HCC827 cells (Fig 1C and 1d, p<0.001 vs. HCC827 cells).

### The decrease in miR-155 in HCC827GR cells was accompanied by an increase in smad2 protein

**Fig 2:**
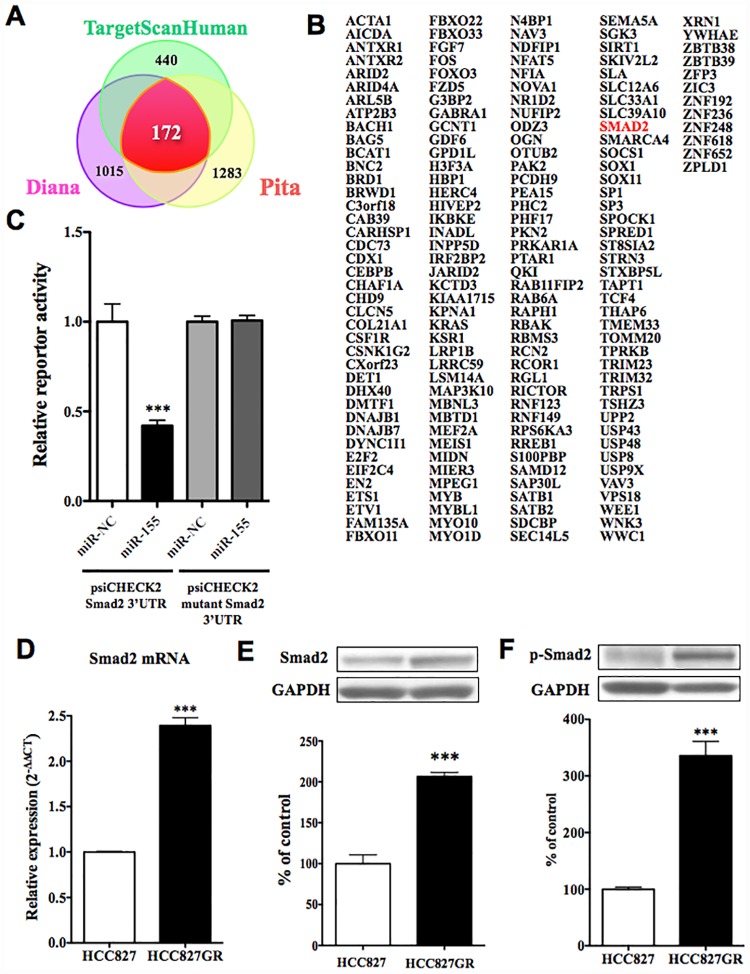
Changes in predicted targeting gene for miR-155 in HCC827GR cells. (A) miR-155 targeting predictions were queried in three target prediction databases. (B) Result of the analysis of 172 predicted targets for miR-155. (C) Assessment of luciferase reporter activity in HEK293 cells co-transfected with expression vector of miR-155 and reporter vector of smad2 3’UTR or mutation smad2 3’UTR. The data represent the relative expression levels of renilla luciferase expression standardized to firefly luciferase (***p<0.001 vs. psiCHECK2 smad2 3’UTR miR-NC). (D) The mRNA expression level of smad2 was quantified by qRT-PCR in HCC827GR cells compared to HCC827 cells (***p<0.001 vs. HCC827 cells). (E) Change in protein levels of smad2 in HCC827GR cells compared to HCC827 cells. Results are shown as the ratio of the density of smad2 to that of GAPDH. Each column represents the mean±S.E.M. of 3 independent experiments (***p<0.001 vs. HCC827 cells). (F) Change in protein levels of phospho-smad2 in HCC827GR cells. Results are shown as the mean±S.E.M. of 3 independent experiments (***p<0.001 vs. HCC827 cells).

With the use of three target-prediction databases, 172 predicted target genes for miR-155 were identified (Fig 2A and 2B). These predicted target genes for miR-155 included smad2, which is a mediator of TGF-ß signaling and thus regulates multiple cellular processes, such as cell proliferation, apoptosis, and differentiation. To confirm direct interactions between miR-155 and smad2, we performed a reporter assay. We generated reporter constructs that contained the 3’UTR, smad2 or mutant constructs downstream of the renilla luciferase gene. Co-transfection of HEK293 cells with 3’UTR of smad2 reporter vector and expression vectors of miR-155 resulted in a significant down-regulation of luciferase activity compared to that in miR-NC (Fig 2C p<0.001 vs. miR-NC). In contrast, the luciferase activities of mutant construct reporter vector and pre-miR-155 mimics did not differ from that of miR-NC. The expression of smad2 mRNA in HCC827GR cells was significantly greater than that in HCC827 cells (Fig 2D, p<0.001 vs. HCC827cells). The decrease in miR-155 expression in HCC827GR cells was accompanied by significant increases in endogenous levels of smad2 protein in HCC827GR cells compared to HCC827 cells (Fig 2E, p<0.001 vs. HCC827 cells). The protein level of phosphorylated smad2 in HCC827GR cells was also increased compared to that in HCC827 cells (Fig 2F, p<0.001 vs. HCC827 cells).

### The decrease in miR-200c in HCC827GR cells was accompanied by an increase in zeb1 protein

**Fig 3:**
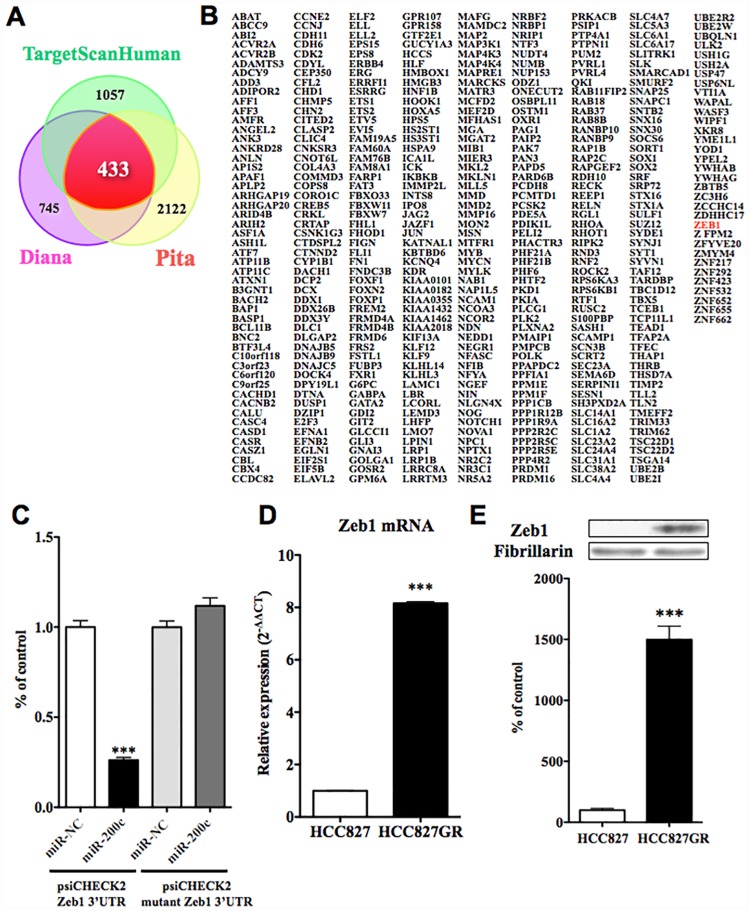
Changes in predicted targeting gene of miR-200c in HCC827GR cells. (A) miR-200c targeting predictions were quantified in three target prediction databases. (B) Result of the analysis of 433 predicted targets for miR-200c. (C) Assessment of luciferase reporter activity in HEK293 cells co-transfected with pre-miR-200c mimics and reporter vector of zeb1 3’UTR or mutation zeb1 3’UTR. The data represent the relative expression level of renilla luciferase expression standardized to firefly luciferase (***p<0.001 vs. psiCHECK2 zeb1 3’UTR miR-NC). (D) The mRNA expression level of zeb1 was quantified by qRT-PCR in HCC827GR cells compared to HCC827 cells (***p<0.001 vs. HCC827 cells). (E) Change in protein levels of zeb1 in HCC827GR cells compared to HCC827 cells. Results are shown as the ratio of the density of zeb1 to that of fibrillarin. Each column represents the mean ± S.E.M. of 3 independent experiments (***p<0.001 vs. HCC827 cells).

While many members of the miR-200 family were decreased in HCC827GR cells, miR-200c was down-regulated most strongly (Fig 1B). With the use of three target-prediction databases, 433 predicted target genes for miR-200c were identified (Fig 3A and 3B).

These predicted target genes for miR-200c included zeb1, which is downstream of smad2. To confirm direct interactions between miR-200c and zeb1, we generated reporter constructs that contained the 3’UTR, zeb1 or mutant constructs downstream of the renilla luciferase gene. Co-transfection of HEK293 cells with 3’UTR of zeb1 reporter vector and pre-miR-200c mimics resulted in a significant down-regulation of luciferase activity compared to that in miR-NC (Fig 3C, p<0.001 vs. miR-NC). In contrast, the luciferase activities of mutant construct reporter vector and pre-miR-200c mimics did not differ from that of miR-NC. The expression of zeb1 mRNA in HCC827GR cells was significantly greater than that in HCC827 cells (Fig 3D, p<0.001 vs. HCC827 cells). The decrease in miR-200c expression in HCC827GR cells was accompanied by a significant increase in the expression level of zeb1 protein in HCC827GR cells compared to HCC827 cells (Fig 3E, p<0.001 vs. HCC827 cells).

### Induction of the Epithelial-to-Mesenchymal Transition (EMT) in HCC827GR cells

**Fig 4:**
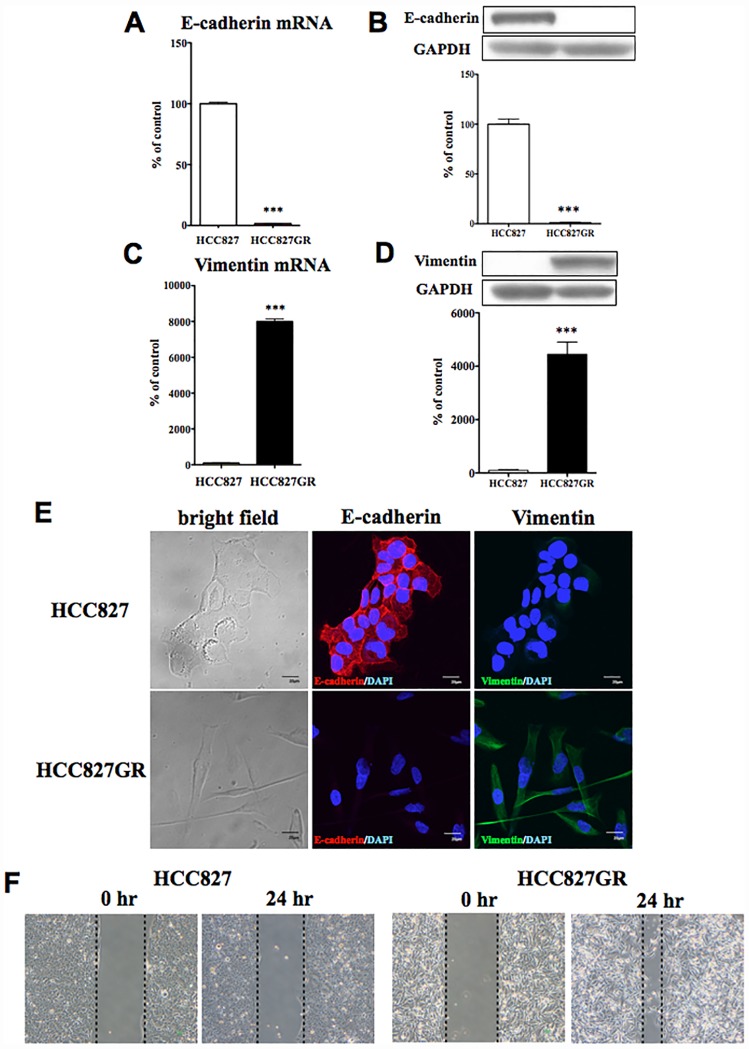
Epithelial-to-mesenchymal transition by chronic *in vitro* treatment with gefitinib. (A,C) The mRNA expression levels of E-cadherin (A) and vimentin (C) were quantified by qRT-PCR in HCC827GR cells compared to HCC827 cells (***p<0.001 vs. HCC827 cells). (B,D) The protein levels of E-cadherin (B) and vimentin (D) were quantified by western blots in HCC827GR cells compared to HCC827 cells. Upper: Representative western blots of E-cadherin (B) and vimentin (D) in cell lysate fraction of HCC827GR cells. Data represent the mean with S.E.M. of 3 independent samples (***p<0.001 vs. HCC827 cells). (E) Immunofluorescent staining for E-cadherin or vimentin in HCC827 and HCC827GR cells. Representative phase-contrast images of HCC827 and HCC827GR cells showing characteristic differences in cell morphology (left). Immunofluorescence staining of E-cadherin (red), an epithelial marker, and DAPI (blue) to identify nuclei, in HCC827 and HCC827GR cells (middle). Immunofluorescence staining of vimentin (green), a mesenchymal marker and DAPI (blue) in HCC827 and HCC827GR cells (right). (F) Wound healing assay in HCC827 and HCC827GR cells. Representative phase-contrast images of HCC827 and HCC827GR cells show characteristic differences in cell migration.

Both smad2 and zeb1 are transcription factors that induce EMT and suppress the transcription of epithelial-cadherin (E-cadherin) by binding to its promoter. Therefore, we performed real-time PCR and western blotting to investigate the changes in mRNA and protein expression of E-cadherin and vimentin, which are both markers of EMT, in HCC827GR cells. The mRNA (Fig 4A) and protein (Fig 4B) levels of E-cadherin were dramatically decreased in HCC827GR cells compared to HCC827 cells (p<0.001 vs. HCC827 cells). In contrast, mRNA (Fig 4C) and protein (Fig 4D) levels of vimentin, which is a mesenchymal marker, were significantly increased in HCC827GR cells compared to HCC827 cells (p<0.001 vs. HCC827 cells).

In a morphological imaging analysis, the morphology of HCC827GR cells was significantly different from that of HCC827 cells (Fig 4E). We noted the specific immunofluorescent staining of E-cadherin and vimentin in HCC827 and HCC827GR cells, respectively, which confirms that the EMT occurred in HCC827GR cells (Fig 4E). We next performed a wound healing assay in HCC827 and HCC827GR cells. As a result, we observed EMT-related migration in HCC827GR cells (Fig 4F).

### Effects of miR-155 and miR-200c inhibitors on HCC827 cells

**Fig 5:**
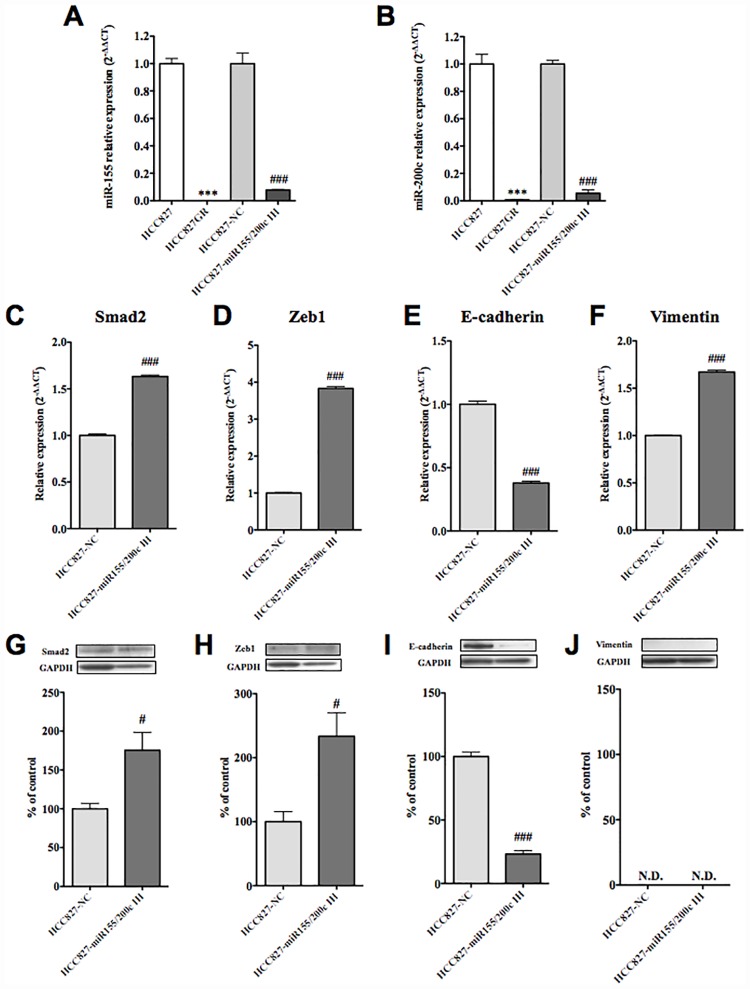
Effects of miR-155 and miR-200c inhibitors on HCC827 cells. (A, B) The HCC827 cells were co-transfected with a specific miR-155 inhibitor and a specific miR-200c inhibitor, or the negative control. Transfection of each inhibitor almost completely abolished the expression of miR-155 (A) and miR-200c (B), respectively (**p<0.01, ***p<0.001 vs. HCC827 or ###p<0.001 vs. HCC827 transfected with a negative control (HCC827-NC)). (C-F) In HCC827 cells transfected with the inhibitors (HCC827-miR155/200c IH), mRNA levels of smad2 (C), zeb1 (D), E-cadherin (E) and vimentin (F) were quantified by qRT-PCR in comparison to the results in the negative control (###p<0.001 vs. HCC827-NC). Each column represents the mean with S.E.M of 6 samples. (G-J) In HCC827 cells transfected with the inhibitors (HCC827-miR155/200c IH), the protein levels of smad2 (G), zeb1 (H), E-cadherin (I) and vimentin (J) were quantified by western blot in comparison to the results in the negative control (#p<0.01, ###p<0.001, vs. HCC827-NC).

We next investigated the effect of dual-inhibition of miR-155 and miR-200c by co-treatment of HCC827 cells with specific inhibitors of both. After co-transfection of HCC827 cells with miR-155 and miR-200c inhibitors (HCC827-miR-155/200c IH), the expression of miR-155 and miR-200c was significantly decreased compared to that in the negative control (HCC827-NC) (Fig 5A and 5B, p<0.01, p<0.001 vs. HCC827-NC). In HCC827-miR-155/200c IH, the expression level of E-cadherin mRNA was significantly decreased (Fig 5E, p<0.001 vs. HCC827-NC), whereas those of vimentin, smad2 and zeb1 mRNA were significantly increased (Fig 5C, 5D and 5F, p<0.001 vs. HCC827-NC). In HCC827-miR-155/200c IH, the protein levels of smad2 and zeb1 were significantly increased (Fig 5G and 5H, p<0.05 vs. HCC827-NC), whereas the protein level of E-cadherin was significantly decreased (Fig 5I, p<0.001 vs. HCC827-NC). In contrast, the protein level of vimentin was not detectable in either type of cell (Fig 5J).

### Changes in histone modifications at the promoter of E-cadherin in HCC827GR cells

**Fig 6:**
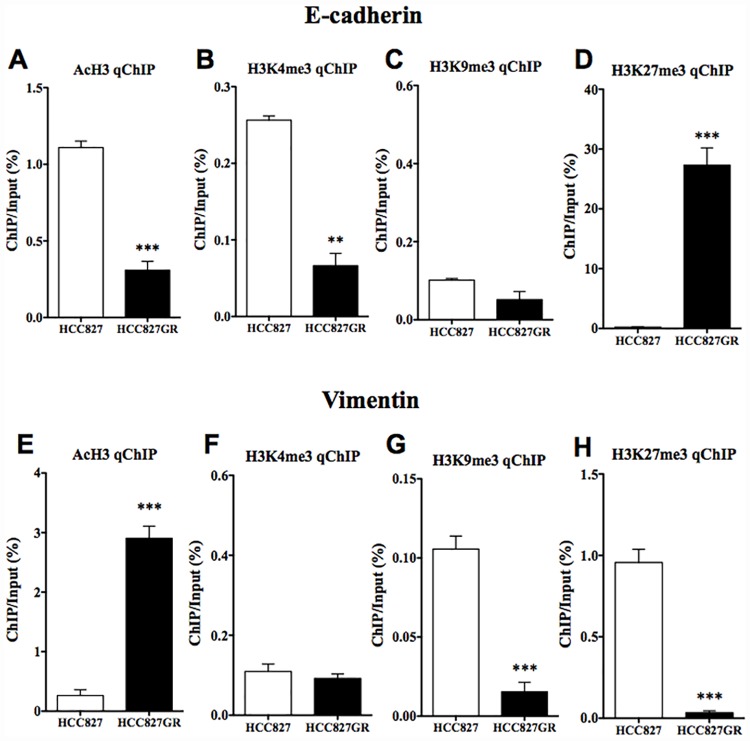
Changes in histone modifications at the E-cadherin or vimentin promoter in HCC827GR cells. (A-D) ChIP-qPCR analysis of (A) AcH3, (B) H3K4me3, (C) H3K9me3 and (D) H3K27me3 at E-cadherin loci was performed in HCC827GR cells compared to that in HCC827 cells. (E-H) ChIP-qPCR analysis of (E) AcH3, (F) H3K4me3, (G) H3K9me3 and (H) H3K27me3 at vimentin loci was performed in HCC827GR cells compared to that in HCC827 cells. The value for ChIP/Input was normalized by that for the internal standard in each control. Each column represents the mean with S.E.M of 3 samples (**p < 0.01, ***p < 0.001 vs. HCC827 cells).

To examine whether the decreased expression of E-cadherin in HCC827GR cells could be regulated by histone modifications, ChIP-qPCR was performed in HCC827GR cells. There was no difference in the level of H3K9me3, which is a repressive histone modification, at the promoter of E-cadherin between HCC827 and HCC827GR cells (Fig 6C). However, significant decreases in AcH3 and H3K4me3, which are active histone modifications, and a significant increase in H3K27me3, which is a repressive histone modification, at the promoter region of E-cadherin were observed in HCC827GR cells compared to HCC827 cells (Fig 6A, 6B and 6D, p<0.01, p<0.001 vs. HCC827 cells).

### Changes in histone modifications at the promoter of vimentin in HCC827GR cells

There was a significant increase in AcH3 at the promoter of vimentin in HCC827GR cells (Fig 6E, p<0.001 vs. HCC827 cells). However, there was no difference in the level of H3K4me3 between the two cell lines (Fig 6F). Furthermore, there were significant decreases in H3K9me3 and H3K27me3 at the promoter region of vimentin in HCC827GR cells compared to HCC827 (Fig 6G and 6H, p<0.001 vs. HCC827 cells).

### Characterization of gefitinib-resistant H1975 cells

Addition of gefitinib (0.001 μM-0.1 μM) to H1975 cells, which are another (*in vivo*) gefitinib-resistant NSCLC cell line, for 2 days did not affect their growth (S1E Fig). According to the results of a sequence analysis, HCC827GR cells had a second point mutation, which was believed to be mainly responsible for drug resistance: nucleotide 2369 C→T mutation in EGFR exon 20, which led to the transition Thr790Met (S1F Fig).

**Fig 7:**
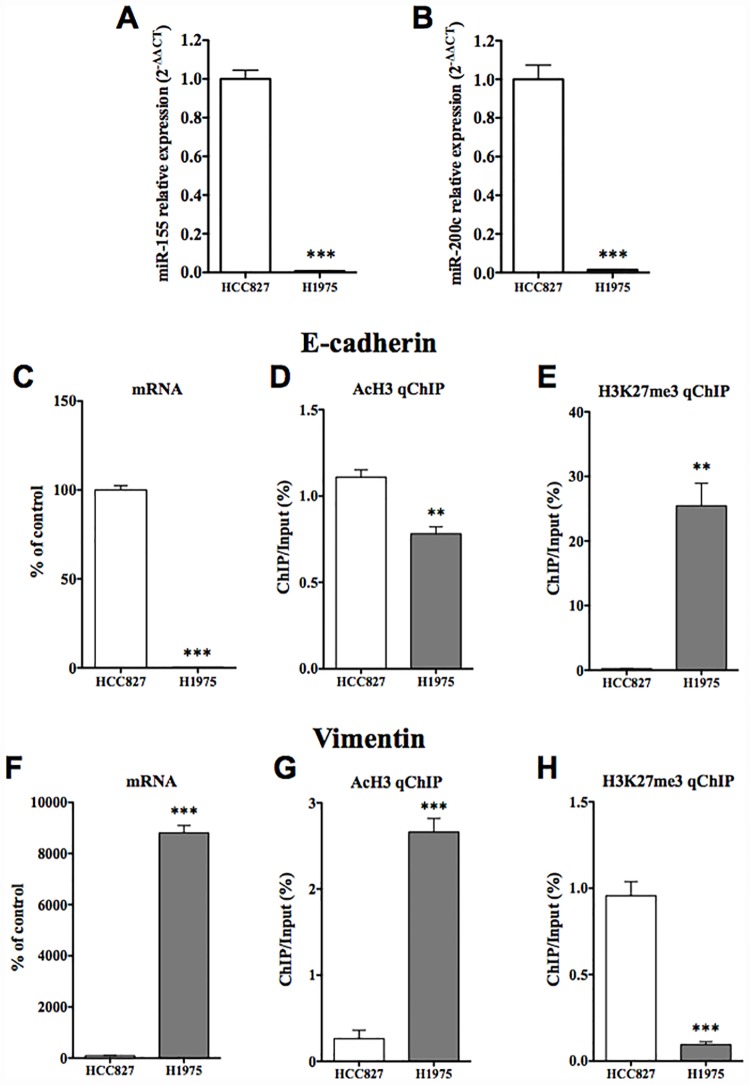
Molecular profiling of another geftinib-resistant H1975 cells compared to HCC827GR cells. (A,B) qRT-PCR for miR-155, miR-200c and RNU44, an internal standard, in HCC827 cells and H1975 cells (***p<0.001 vs. HCC827 cells). (C,F) The mRNA expression levels of E-cadherin (C) and vimentin (F) were quantified by qRT-PCR in H1975 cells compared to HCC827 cells (***p<0.001 vs. HCC827). Each column represents the mean with S.E.M of 6 samples. (D,E) ChIP-qPCR analysis of AcH3 (D) and H3K27me3 (E) at E-cadherin loci was performed in H1975 cells and the results were compared to those in HCC827 cells. (G,H) ChIP-qPCR analysis of AcH3 (G) and H3K27me3 (H) at vimentin loci was performed in H1975 cells and the results were compared to those in HCC827 cells. The value for ChIP/Input was normalized by that for the internal standard in each control. Each column represents the mean with S.E.M of 3 samples (**p < 0.01, ***p < 0.001 vs. HCC827 cells).

The expressions of both miR-155 and miR-200c were significantly decreased in H1975 cells compared to those in HCC827 cells (Fig 7A and 7B, p<0.001 vs. HCC827 cells). In H1975 cells, the mRNA of E-cadherin was significantly decreased compared to that in HCC827 cells (Fig 7C, p<0.001 vs. HCC827 cells), while the mRNA of vimentin was significantly increased compared to that in HCC827 cells (Fig 7F p<0.001 vs. HCC827 cells). Under these conditions, there was a significant decrease in AcH3 at the promoter of E-cadherin in H1975 cells (Fig 7D, p<0.01 vs. HCC827 cells), and a dramatic increase in H3K27me3 at the promoter region of E-cadherin was observed in H1975 cells compared to HCC827 cells (Fig 7E, p<0.001 vs. HCC827 cells). On the other hand, there was a significant increase in AcH3 at the promoter of vimentin in H1975 cells compared to that in HCC827 cells (Fig 7G, p<0.001 vs. HCC827 cells). In contrast, the level of H3K27me3 at the promoter of vimentin was significantly decreased in H1975 cells (Fig 7H, p<0.001 vs. HCC827 cells).

## Discussion

It has been reported that patients who suffer from NSCLC with an in-frame deletion (E746-A750) in EGFR exon 19 are highly responsive to gefitinib [4, 15]. However, patients who initially benefit from gefitinib later acquire resistance to treatment and then experience a more rapid progression of symptoms [5]. Such acquired resistance to gefitinib has been associated with a secondary T790M mutation in EGFR exon 20 [3, 4]. In our study, gefitinib-sensitive NSCLC cells, HCC827 cells, exhibited a gefitinib concentration-dependent decrease in cell viability and had deletional mutations (E746-A750) in EGFR exon 19, as previously reported. After HCC827 cells were re-exposed to an increasing concentration of gefitinib, we finally obtained gefitinib-resistant cells that had a secondary T790M mutation in EGFR exon 20, as previously reported. We defined these gefitinib-resistant HCC827 cells as HCC827GR cells.

miRNAs that are up- or down-regulated are known to play key roles in malignancies in various tissues, such as lung [7], breast [8], liver [9], colon and rectum [10] and prostate [11]. In the present study, we investigated whether chronic treatment of gefitinib-sensitive NSCLC cells with gefitinib could change the expression of miRNAs, and in turn induce cell transformation associated with gefitinib-resistance.

First, we performed a miRNA-specific microarray analysis to compare the expression profiles of mRNAs encoding 768 miRNAs in HCC827 and HCC827GR cells. Using this miRNA-specific microarray analysis, we found that miR-155, miR-200c and other members of the miR-200 family, including miR-200a, miR-200b, miR-141 and miR-429, were dramatically down-regulated in HCC827GR cells compared to HCC827 cells. Furthermore, we also found very low expression levels of miR-155 and miR-200c in another gefitinib-resistant NSCLC cell line H1975.

The EMT is an important process in tumor metastasis and cancer invasiveness [16]. In this process, cells lose their epithelial characteristics and become able to migrate into surrounding tissue. In the present study, through the use of three target-prediction databases, we identified 172 and 433 predicted target genes for miR-155 and miR-200c, respectively. The gene list included smad2 and zeb1, which are both key players in EMT, as the targets for miR-155 and miR-200c, respectively. Zeb1 is downstream of smad2 [17], which mediates TGF-ß signaling [18]. To confirm direct interactions between miR-155 and smad2, and between miR-200c and zeb1, we performed a 3’UTR reporter assay, and found that miR-155 and miR-200c could directly control the expression of smad2 and zeb1, respectively. Through the use of western blotting, we found that the reductions in miR-155 and miR-200c observed in HCC827GR cells, which can modulate the protein translation of smad2 and zeb1, respectively, were accompanied by dramatic increases in protein levels of both smad2 and zeb1 in HCC827GR cells compared to HCC827 cells. It has been reported that TGF-ß signaling plays an important role in EMT [19]. In particular, TGF-ß activates the receptor complex, and smad2 and smad3 are activated via direct phosphorylation of their C-termini. They then form trimers with smad4 that translocate into the nucleus, where they participate in the binding of DNA to transcription factors, which can either promote or suppress the transcription of a target gene. Thus, smad2 and smad3 can act together with smad4 to regulate transcription under the influence of TGF-ß [20]. In agreement with this important function of smads, the EMT occurs via a well-regulated sequence of transcription that involves members of the bHLH family, like snail, slug, and zeb [21,22,23]. Their expressions are known to be induced in response to TGF-ß, either through a smad-dependent mechanism or indirectly through the activation of other transcription factors or the relief of repression. These transcriptional factors act as repressors of E-cadherin, which is an epithelial cell marker [24]. In support of the notion that an EMT event could occur in gefitinib-resistant NSCLC cells, the level of E-cadherin was greatly decreased in HCC827GR and H1975 cells, whereas the level of vimentin, which is dominantly expressed in mesenchymal cells, was dramatically increased in these cells. Therefore, we hypothesized that EMT would occur during the development of gefitinib-resistance in NSCLC cells through reductions in miR-155 and members of the miR-200 family, and increases in smad2 and zeb1.

Next, we investigated whether dual-inhibition of miR-155 and miR-200c by co-transfection of HCC827 cells with specific inhibitors of both could mimic HCC827GR- and H1975-like characteristics. In this study, both mRNA and protein levels of smad2 and zeb1 were significantly increased, and mRNA and protein levels of E-cadherin were dramatically decreased, indicating that the dual-inhibition of miR-155 and miR-200c in HCC827 cells induces an HCC827GR-like loss of the epithelial profile. In contrast, although the mRNA level of vimentin was significantly increased by such dual-inhibition, the protein level of vimentin was not detectable in HCC827 cells treated with or without miR155/200c inhibitors. These findings suggest that chronic treatment with gefitinib depletes the expression level of miR-155 and miR-200c in HCC827 cells and this is associated with a loss of the epithelial profile and the appearance of a primary mesenchymal state. Li et al. reported that the overexpression of miR-200c in NSCLC cells reverses the mesenchymal phenotype of cells to the epithelial phenotype by targeting zeb1 [25]. They also found that upregulation of miR-200c increased gefitinib sensitivity in the EGFR non-mutation NSCLC cell line A549, but not in H1975 cells, which exhibit EGFR-T790M-mutation, whereas blocking the expression of miR-200c in PC9 cells, which are NSCLC cells that have both EGFR-L858R-mutation and a deletional mutation (E746-A750) in EGFR exon 19, was associated with slight resistance to gefitinib. On the other hand, Chiu et al. observed a significant increase in miR-155 in gefitinib-resistant PC9 cells [26]. In the present study, chronic treatment with gefitinib in HCC827 cells, which do not exhibit an EGFR-L858R-mutation but do show an EGFR-E746-A750 deletion (data not shown), established gefitinib-resistance along with the depletion of both miR-155 and miR-200c, while the chronic inhibition of both miR-155 and miR-200c in HCC827 cells decreased gefitinib sensitivity (S2A Fig). Furthermore, this dual-inhibition in HCC827 cells without gefitinib was not associated with a secondary T790M mutation in EGFR exon 20 (S2B Fig). Taken together, our results suggest that, although the mutation-dependent mechanism would be complex, the depletion of both miR-200c and miR155 by chronic treatment of NSCLC cells with gefitinib may act as a trigger for the EMT and contribute to gefitinib-resistance independent of a secondary T790M mutation in EGFR.

To further understand the mechanism of EMT during the development of gefitinib-resistance in NSCLC cells, we investigated whether epigenetic modifications could be involved in EMT. Epigenetic mechanisms enhance or suppress gene expression without changing the primary DNA sequence. Epigenetic mechanisms can be dynamic and responsive to changes in experience, and thus represent a complex interplay between an organism and its environment. The acetylation of most histone subunits, at any of several lysine (Lys) residues, typically promotes gene transcription, while histone methylation can either repress or activate gene transcription depending on the amino acid residue undergoing methylation. For example, methylation of Lys9 or Lys27 of histone H3 is usually associated with gene repression, whereas methylation of Lys4, Lys36, or Lys79 of H3 is usually associated with gene activation. The present key finding related to the decrease in E-cadherin was that both a significant decrease in the active histone modification and a significant increase in the repressive histone modification at the promoter region of E-cadherin were observed in both HCC827GR and H1975 cells. In contrast, both cells showed a dramatic increase in the active histone modification and a significant decrease in the repressive histone modification at the promoter region of vimentin. These findings suggest that epigenetic modifications at the promoter regions of E-cadherin and vimentin could be responsible for the gefitinib-induced EMT of NSCLC cells.

## Conclusion

In conclusion, chronic treatment of NSCLC cells with gefitinib depletes the expression of both miR-155 and miR-200c. This depletion of miR-155 and miR-200c in NSCLC cells may lead to the EMT along with histone modification and contribute to gefitinib-resistance independent of a secondary EGFR-T790M-mutation.

## Ethics/Consent

Our manuscript does not report data collected from humans or animals. Also, this was not a clinical trial. The cell lines used in the present study were basically purchased from the manufacturer.

## Supporting information

S1 Fig Effects of gefitinib on the growth of HCC827, HCC827GR and H1975 cells. (A) Viability of HCC827cells treated with gefitinib. HCC827 cells were incubated with gefitinib (0.001–0.1μM) for 2 days, and then cell viability was measured (***p<0.001 vs. non-treated group). (B) Sequence analysis of EGFR exon 19 in HCC827 cells. HCC827 cells had an in-frame deletion (E746-A750) in EGFR exon 19. (C,E) Viability of HCC827GR cells or H1975 cells treated with gefitinib. Cells were incubated with gefitinib (0.001–0.1μM) for 2 days, and then cell viability was measured. (D,F) Sequence analysis of EGFR exon 20 in HCC827GR cells (D) and H1975 cells (F). HCC827cells and H1975 cells had a T790M-mutation in EGFR exon 20.
(TIFF)

S2 Fig Effects of miR-155 and miR200c inhibitors on the gefitinib-induced changes in HCC827 cell viability. (A) Cell viability following treatment with gefitinib (0.001–1 μM) in HCC827 cells transfected with negative control or HCC827 cells co-transfected with miR-155 and miR-200c inhibitors. The inhibition of miR-155 and miR-200c in HCC827 cells slightly, but significantly decreased gefitinib sensitivity (*p<0.05 vs. HCC827-NC group). (B) Sequence analysis of EGFR exon 20 in HCC827 cells with miR-155 and miR-200c inhibitors. The inhibition of miR-155 and miR-200c in HCC827 cells without gefitinib did not produce a secondary T790M mutation in EGFR exon 20.
(TIFF)

S1 Table Probe sequences used for qRT-PCR for miRNA. (TIFF)

S2 Table Primary antibody. (TIF)

S3 Table Primer sequences used for dual luciferase 3’UTR-reporter assays. (TIF)

S4 Table Primer sequences used for qRT-PCR. (TIF)

S5 Table Primer sequences used for ChIP-qPCR. (TIF)

S1 File Supplementary materials and methods. (DOCX)

## Abbreviations

AcH3: acetyl-Histone H3
E-cadherin: Epithelial cadherin
EGFR: epidermal growth factor receptor
EMT: epithelial-to-mesenchymal transition
H3K27me3: trimethyl-Histone H3 Lys27
H3K4me3: trimethyl-Histone H3 Lys4
H3K9me3: trimethyl-Histone H3 Lys9
miRNA: microRNA
NSCLC: non-small-cell lung cancer
Smad2: Smad family member 2
Zeb1: zinc finger E-box binding homeobox 1
