# Supplementary figures and images for: Chronic treatment of non-small-cell lung cancer cells with gefitinib leads to an epigenetic loss of epithelial properties associated with reductions in microRNA-155 and -200c
Source: PLoS One. 2017 Feb 22;12(2):e0172115. doi: 10.1371/journal.pone.0172115 (PMC5321411; doi:10.1371/journal.pone.0172115)

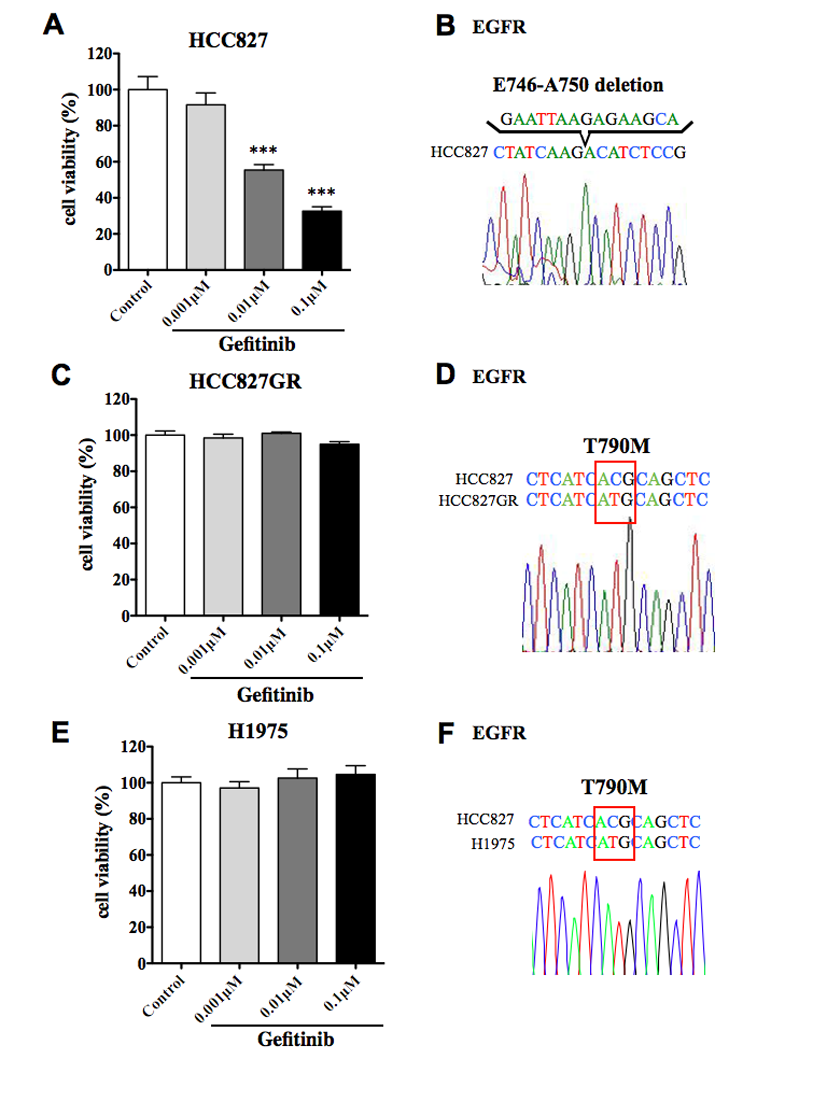

Supplement: S1 Fig — (A) Viability of HCC827cells treated with gefitinib. HCC827 cells were incubated with gefitinib (0.001–0.1μM) for 2 days, and then cell viability was measured (***p<0.001 vs. non-treated group). (B) Sequence analysis of EGFR exon 19 in HCC827 cells. HCC827 cells had an in-frame deletion (E746-A750) in EGFR exon 19. (C,E) Viability of HCC827GR cells or H1975 cells treated with gefitinib. Cells were incubated with gefitinib (0.001–0.1μM) for 2 days, and then cell viability was measured. (D,F) Sequence analysis of EGFR exon 20 in HCC827GR cells (D) and H1975 cells (F). HCC827cells and H1975 cells had a T790M-mutation in EGFR exon 20. (TIFF) [file pone.0172115.s001.tiff]

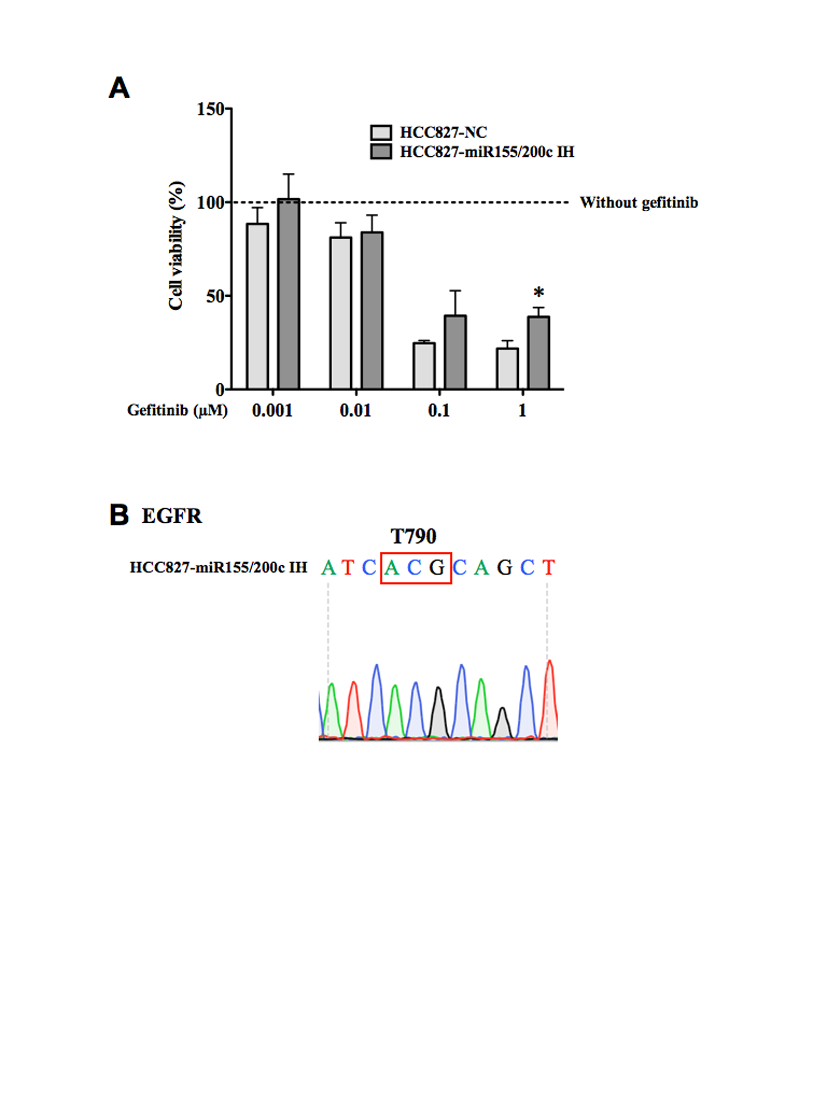

Supplement: S2 Fig — (A) Cell viability following treatment with gefitinib (0.001–1 μM) in HCC827 cells transfected with negative control or HCC827 cells co-transfected with miR-155 and miR-200c inhibitors. The inhibition of miR-155 and miR-200c in HCC827 cells slightly, but significantly decreased gefitinib sensitivity (*p<0.05 vs. HCC827-NC group). (B) Sequence analysis of EGFR exon 20 in HCC827 cells with miR-155 and miR-200c inhibitors. The inhibition of miR-155 and miR-200c in HCC827 cells without gefitinib did not produce a secondary T790M mutation in EGFR exon 20. (TIFF) [file pone.0172115.s002.tiff]

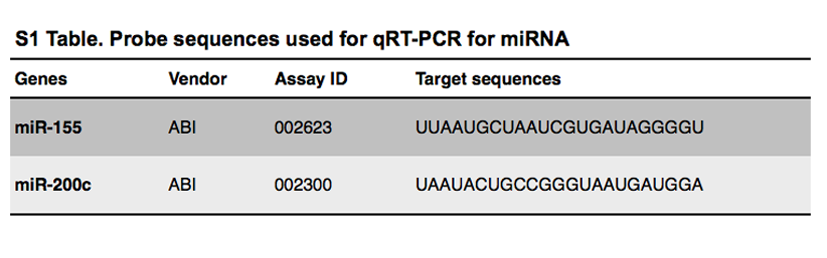

Supplement: S1 Table — (TIFF) [file pone.0172115.s003.tiff]

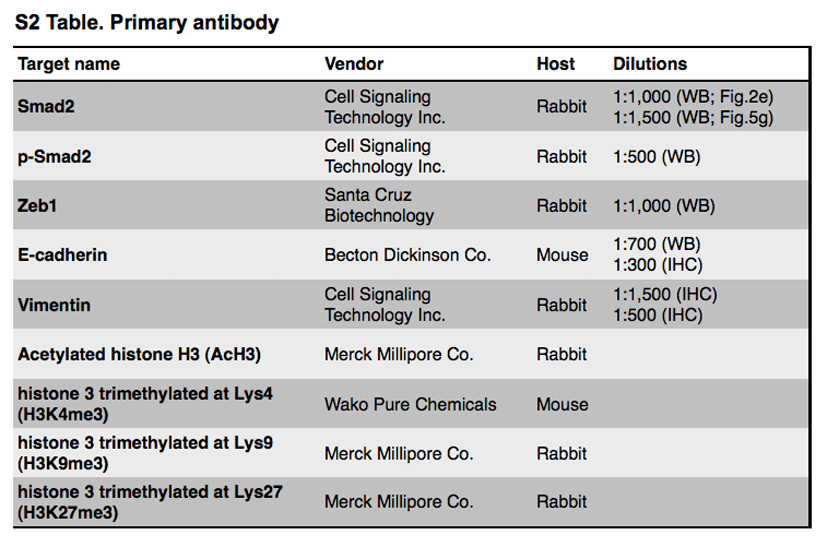

Supplement: S2 Table — (TIF) [file pone.0172115.s004.tif]

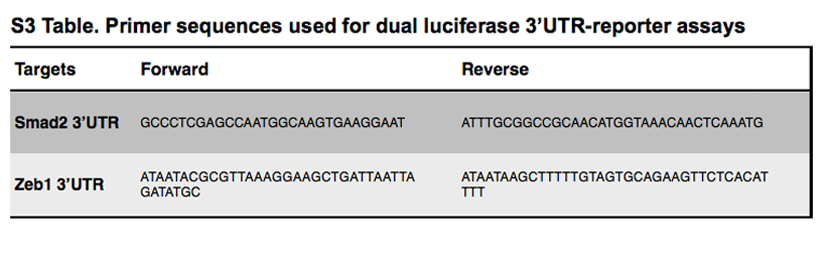

Supplement: S3 Table — (TIF) [file pone.0172115.s005.tif]

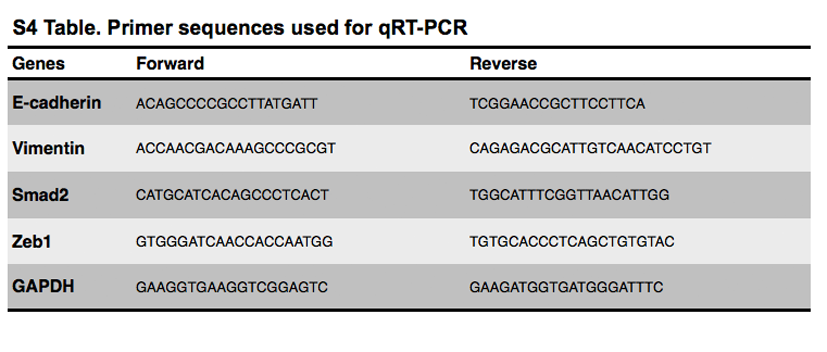

Supplement: S4 Table — (TIF) [file pone.0172115.s006.tif]

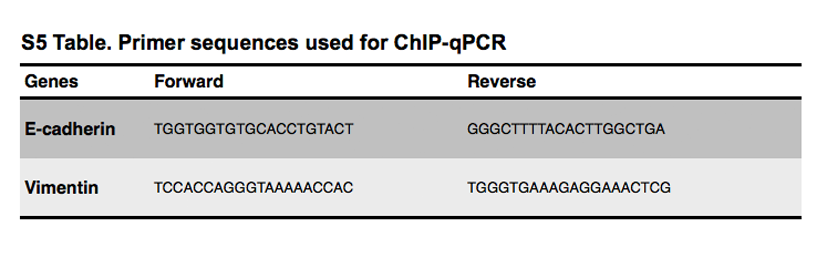

Supplement: S5 Table — (TIF) [file pone.0172115.s007.tif]
