# Supplementary material for: Chronic treatment of non-small-cell lung cancer cells with gefitinib leads to an epigenetic loss of epithelial properties associated with reductions in microRNA-155 and -200c
Source: PLoS One. 2017 Feb 22;12(2):e0172115. doi: 10.1371/journal.pone.0172115 (PMC5321411; doi:10.1371/journal.pone.0172115)
Supplement: S1 File — (DOCX) [file pone.0172115.s008.docx]

**S1 Methods**

**Cell viability assay**

Cell viability was determined by the 3-(4,5-dimethylthiazol-2-yl)-2,5- diphenyl-tetrazolium bromide (MTT) assay. 20 μL of MTT solution (5 mg/mL) was added to each well of the culture medium. After incubation for another 2 h, the medium was removed, and 100 μL of DMSO was added to resolve formazan crystals. Optical density was measured using a luminometer (Glomax, Promega, WI, USA) at an absorption wavelength of 600 nm. In each experiment, three replicates were prepared for each sample. The proportion of living cells was determined based on the difference in absorbance between samples and controls.

**Mutation analysis**

EGFR exon 19 and 20 were amplified by PCR using gene-specific primers

(EGFR exon 19 (L747-S752del); sense:5'-GTTAAAATTCCCGTCGCTATC-3' antisense: 5'-GGACATAGTCCAGGAGGCAG-3', EGFR exon 20 (T790M); sense :5’-GAAGCAACATCTCCGAAAGC-3’antisense:5’-AGGTCATCAACTCCCAAACG-3’). The PCR products were gel-purified using a QIAEXII Gel Extraction kit (Qiagen, Valencia, CA, USA). PCR products were subcloned into the pGEM-T easy cloning vector (Invitrogen^TM^ Life Technologies Co.) and sequenced. The resulting PCR products were sequenced using the Big Dye Terminator version 3.1 Cycle Sequencing Ready Reaction Kit (Applied Biosystems, Carlsbad, CA, USA) following the manufacturer’s protocol.
